# Detection of lymphangiogenesis in non-small cell lung cancer and its prognostic value

**DOI:** 10.1186/1756-9966-28-21

**Published:** 2009-02-16

**Authors:** Jian-guo Sun, Yan Wang, Zheng-tang Chen, Wen-lei Zhuo, Bo Zhu, Rong-xia Liao, Shao-xiang Zhang

**Affiliations:** 1 Cancer Institute of People's Liberation Army, Xinqiao Hospital, Third Military Medical University, Chongqing, 400037, PR China; 2 Department of Biochemistry and Molecular Biology, Third Military Medical University, Chongqing, 400038, PR China; 3 Department of Anatomy, College of Medicine, Third Military Medical University, Chongqing, 400038, PR China

## Abstract

**Background:**

Our aim was to detect lymphatic endothelial marker podoplanin, lymphatic vessel endothelial hyaluronan receptor-1 (LYVE-1) and vascular endothelial growth factor receptor-3 (VEGFR)-3 and study the prognostic relevance of lymphangiogenesis in non-small cell lung cancer (NSCLC).

**Materials:**

82 paraffin-embedded tissues and 40 fresh frozen tissues from patients with NSCLC were studied. Tumor samples were immunostained for the lymphatic endothelial markers. Lymphangiogenesis was assessed by immunohistochemical double stains for Podoplanin and Ki-67. The prognostic relevance of lymphangiogenesis-related clinicopathological parameters in NSCLC was evaluated.

**Results:**

We found that the number of podoplanin positive vessels was correlated positively with the number of LYVE-1 positive vessels. Most of VEGFR-3 positive, few of LYVE-1 positive and none of podoplanin positive vessels were blood vessels. Peritumoral lymphatic vessel density (ptLVD), pathologic stage, lymph node status, lymphatic vessel invasion (LVI), vascular endothelial growth factor-C (VEGF-C) expression and Ki-67 index of the endothelium cells of the micro lymphatic vessels (Ki67%) were associated significantly with a higher risk of tumor progress. ptLVD, pathologic stage, lymph-node metastasis and Ki67% were independent prognostic parameters for overall survival.

**Conclusion:**

Podoplanin positive ptLVD might play important roles in the lymphangiogenesis and progression of NSCLC. Patients with high podoplanin+ ptLVD have a poor prognosis.

## Background

The major cause of death from malignant tumors including non-small cell lung cancer (NSCLC) is dissemination of the primary tumor, leading to formation of metastases. Spread to regional lymph nodes is often the first step of generalization. Thus, the presence of lymph node metastasis represents a major criterion for evaluating the prognosis of NSCLC patients. Tumor-associated lymphangiogenesis are considered as the main route for lymphatic metastasis. And lymphovascular invasion (LVI) of tumor cells is a prerequisite for the dissemination via the lymphatic system. However, Studies of lymphatic vessels and lymphogenic metastasis have been hampered by the lack of specific lymphatic markers. Recently several markers for normal and tumor-associated lymphatic vessels have provided tools for a detailed analysis of lymphangiogenesis in human lung cancers. These markers include vascular endothelial growth factor C and D (VEGF-C, VEGF-D) [1,2], vascular endothelial growth factor receptor-3 (VEGFR-3) [3-6], the lymphatic vessel endothelial hyaluronan receptor-1 (LYVE-1) [7] and glomerular podocyte membrane mucoprotein podoplanin [8]. Podoplanin had been speculated as a specific lymphatic endothelial marker in many solid tumors including breast cancer, cervical cancer, Ovarian Cancer and NSCLC [9,10]. However, there are some contradictory results between different studies and many problems to be clarified. For example, VEGFR-3 is expressed not only in lymphatic endothelium in normal adult tissue, but also in vascular endothelium in tumor tissue. Therefore, using VEGFR-3 as a marker of tumor lymph vessel may lead to loss of accuracy in lymphatic vessel density (LVD) counting [11]. LYVE-1 was thought to be restricted to lymphatic vessels [12]. However, LYVE-1 was also found in normal hepatic blood sinusoidal endothelial cells and macrophage [13,14]. The specificity of LYVE-1 for lymphatic endothelial cells (LECs) has been questioned by some investigators [15]. Futhermore, Padera [16] showed that approximately 10% of LYVE-1+ vessels were indeed blood vessels, suggesting that LYVE-1 alone is not suitable for the detection of functional lymphatic vessels. Until recently, tumorologists have recognized podoplanin as the most specific marker for lymphatic endothelium. And a double immunostaining with the D2–40 and anti-Ki67 monoclonal antibody is used as the standard method for the assessment of lymphangiogenesis in solid tumors[17]. Thus, the aim of this study was to detect Lymphangiogenesis and find the relationship between clinicopathological parameters, such as LVD, lymph-node metastasis, VEGF-C, LVI, pathological stage, and prognostic factor in NSCLC.

## Methods

### Patients and tissues

This retrospective study included 82 patients with NSCLC who underwent either lobectomy or pneumonectomy at Xinqiao Hospital between January 1995 and November 2004. All of these patients have complete clinical and pathological records. None of the patients received presurgical radio- or chemotherapy before operation. Follow-up was made to August 31, 2005, by phone call, letter inquiry and visiting census register agency. During the follow-up period, there were 35 patients still alive and 47 deaths. Patients who were lost to follow up or died for noncancer-related reasons were excluded. Pathological stage was reevaluated and determined with the present TNM classification as revised in WHO 2004 classification criteria. Formalin-fixed, paraffin-embedded NSCLC tissues were retrieved from the files of our pathology department. Tissue blocks containing a representative fraction of the tumor and the tumor-lung parenchyma interface were used. Operative tissues embedded with paraffin from the 82 patients with NSCLC. In addition, the fresh frozen operation tissues of 40 NSCLC patients from Xinqiao and Daping hospital were used for LYVE-1 immunohistochemistry and H&E staining (LYVE-1 expression was only on the fresh frozen sections, not on paraffin sections). The study was approved by the Ethics Committee (Faculty of Medicine, Third Military Medical University).

### Immunostaining for LYVE-1, CD31, VEGFR-3, podoplanin, VEGF-C and Vessel Counts

Immunohistochemical stainings were done on tissues fixed in 10% neutral buffered formalin and embedded in paraffin. Paraffin sections (5 μm) were dewaxed and rehydrated. For light microscopy, peroxidase was quenched with methanol and 3% H_2_O_2 _for 15 minutes. Antigen retrieval was done in 0.1 mol/L citrate buffer (pH = 6) in an 800W microwave for 15 minutes (the step was omitted in fresh frozen section staining). After washing in PBS, the following primary antibodies were used: rabbit polyclonal anti-human LYVE-1 (10 μg/ml, Angiobio Co, USA), rabbit monoclonal anti-human podoplanin (1:100, Angiobio Co, USA), mouse monoclonal anti-human CD31 (ready to use, Zhongshan, Beijing), rabbit polyclonal anti-human VEGFR-3, VEGF-C (ready to use, Zhongshan, Beijing). All primary and secondary IgGs were diluted in PBS. Isotypic controls were performed for monoclonal as well as use of non immune serum for polyclonal antibodies (same concentrations as the test antibodies). Determination of LVD (assessed by immunostaining for podoplanin, LYVE-1, VEGFR-3) and CD31 microvessel density (MVD) was performed as suggested by Weidner [18]. Briefly, the immunostained sections were first scanned at a low magnification (40×), and the areas with the greatest number of microvessels (vessel "hot spots") were selected for further evaluation. The microvessel count was then determined by counting all immunostained vessels in five separate hot spots at a high magnification (×200). The average number of LVD or MVD in the five selected vessel hot spots was then calculated. In immunostainings for CD31, podoplanin, LYVE-1 and VEGFR-3, any positive cell clusters were considered as endothelial cells and countable microvessels. LVI was considered evident if at least one tumor cell cluster was clearly visible inside the podoplanin-stained vascular space [19]. Peritumoral lymphatic vessels were defined as LYVE-1/podoplanin/VEGFR-3-positive vessels within an area of 100 μm from the tumor border. Intratumoral lymphatic vessels were defined as LYVE-1/podoplanin/VEGFR-3-positive vessels located within the tumor mass and not confined by invagination of normal tissue [20].

### Double immunostaining with podoplanin and Ki-67

Immunohistochemical double stains for Podoplanin and Ki67 were done on serial sections according to Van den Eynden's method [21]. Podoplanin and Ki-67 was stained by D2–40 and anti-Ki67 monoclonal antibody, respectively. (Angiobio & Beijing Zhongshan Jinqiao Biotechnology Co., respectively) Histastain™-DS double immunostaining kit was purchased from Zymed. In brief, sections were first incubated with primary antibody, *i.e*. podoplanin (dilution 1:200), and biotinized secondary antibody, which was visualized with the Envision + dual link system (Dakocytomation, Carpinteria, CA, USA). A second primary antibody, i.e. Ki67 (dilution 1:100) was then applied and visualized with the Envision G/2 system/AP (Dakocytomation, Carpinteria, CA, USA). Micro lymphatic vessels were brownish yellow after staining, while the nucleus of the proliferating endothelium cells of the micro lymphatic vessels appeared red. Ki-67 index of the endothelium cells of the micro lymphatic vessels (Ki67%) was calculated according to Wulff et al [22].

### Statistical Analysis

Correlations between podoplanin, VEGFR-3, LYVE-1 and the vessel numbers as continuous variables were used to assess CD31-positive vessel counts with the Spearman rank correlation test. Categorical data were compared by the χ^2 ^or Fishers' exact probability test. Distribution was normal or with Mann Whitney U test if the sample distribution was asymmetrical. The relationship between lymph vessel variables and lymph node status was analyzed by one-way ANOVA, followed by the Neuman-Keuls test. Overall survival intervals were determined as the time period from initial diagnosis to the time of death. Overall survival analyses were done using the Kaplan-Meier method. The comparison between survival functions for different strata was assessed with the log-rank statistic. Multivariate analysis of prognostic factors was done using Cox's regression model. Differences were considered significant when *P *≤ 0.05. All statistical analyses were done using the statistical package spss13.0.

## Results

### CD31, VEGFR-3, LYVE-1, VEGF-C Expression in NSCLC

**Figure 1:**
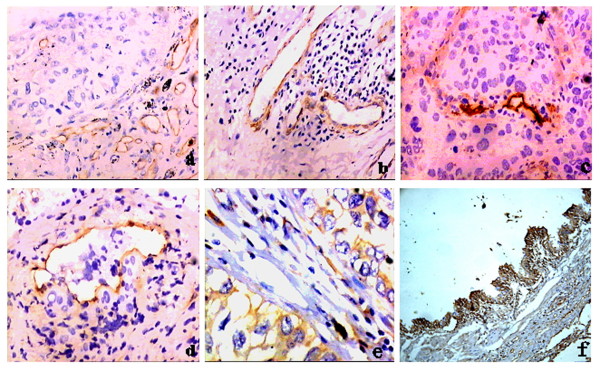
**Immunohistochemical analysis of different markers**.

Numerous intratumoral and peritumoral vessels could be observed in each NSCLC tumor irrespective of histologic grade and pathologic stage. CD31 was positive in endothelial cell plasma in micro vessels, appeared yellow granular. Micro vessels of tumor tissues were mainly located at intra-tumor and peritumoral area. However, large blood vessels with muscular coat were also positive stained for CD31 (Fig. 1a). VEGFR-3 showed an expression similar to CD31. VEGFR-3 positive vessels included not only dilated and irregular thin-walled lymphatic vessels, but also blood vessels containing erythrocytes and large blood vessels with smooth muscle (Fig. 1b). LYVE-1 was positively stained in endothelial cell plasma and plasma membrane in micro vessels, appeared yellow granular (Fig. 1c). However, few LYVE-1 positive vessels were large blood vessels with smooth muscle, and tumor embolus were observed in their muscular layer and lumen (Fig. 1d). VEGF-C positive substance in tumor tissue was yellow fine granular, mainly located in tumor cell plasma. Positive cells were dispersed, limited locally or in small patches (Fig. 1e). In the para-tumor normal bronchia, VEGF-C expression was dispersed in columnar epithelium cells (Fig. 1f).

### Podoplanin Expression in NSCLC

**Figure 2:**
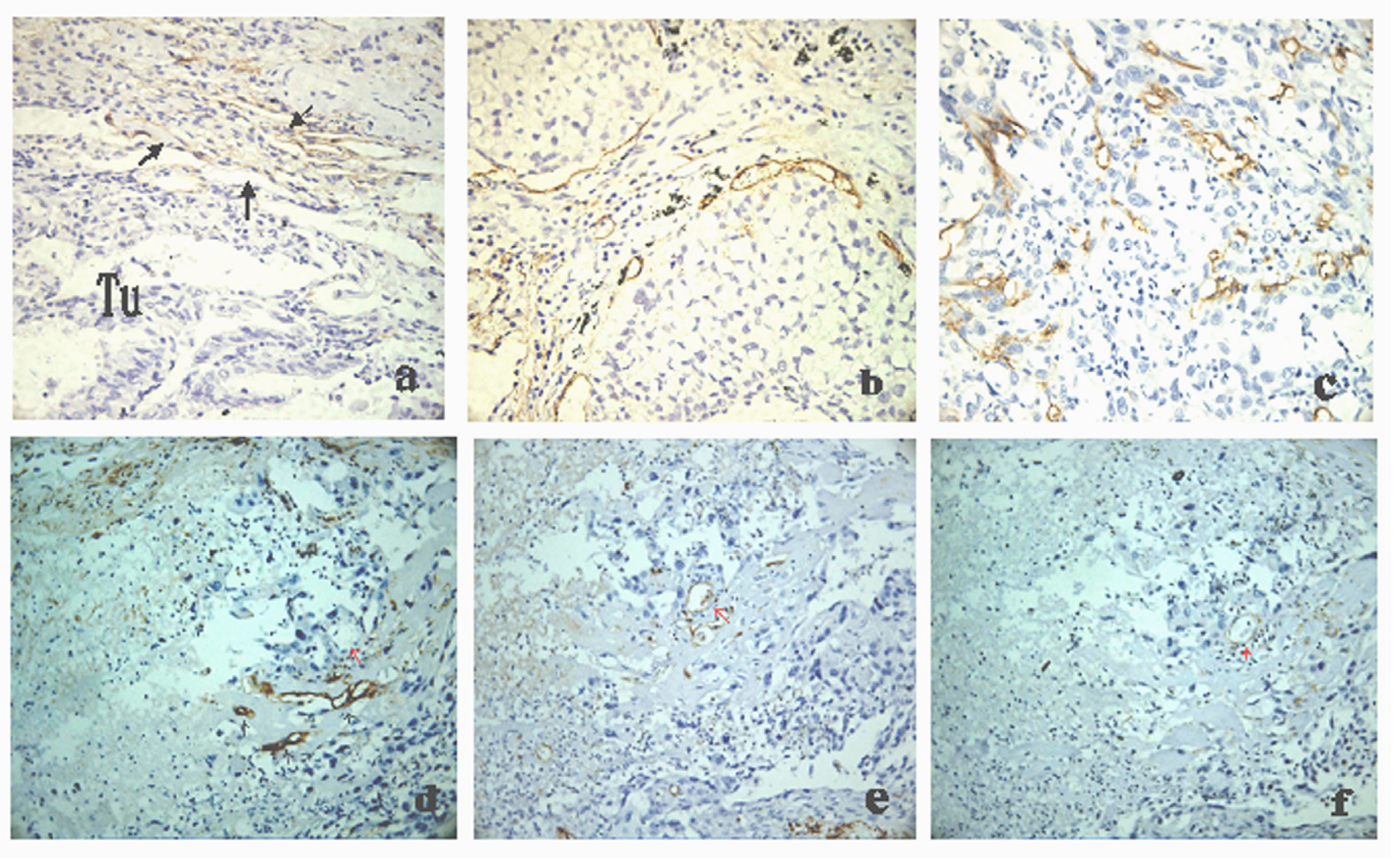
**Immunostaining for podoplanin in nsclc tissues**.

Podoplanin expression was mainly present in thin-walled (lymphatic) structures. Podoplanin was positive in endothelial cell plasma in thin-walled lymph vessel, appeared yellow granular. Podoplanin-positive lymph vessels were commonly located around the tumor or at the tumor boundary, mainly located at adenocarcinoma interstitium, peri-tumor area of squamous cell carcinoma and interstitium of large cell lung cancer (Fig. 2a, b, c). 4 cases of squamous cell carcinoma also demonstrated podoplanin expression in cancer cell plasma (data not shown). Moreover, we cut serial sections of lung cancer tissue, and stained them with podoplanin, CD31 and VEGFR-3, respectively. The red arrow in Fig. 2d indicates podoplanin-negative blood vessels. Black arrow in Fig. 2d indicates podoplanin-positive lymph vessel. While in Fig. 2e and 2f, the same region was positively stained for CD31 and VEGFR-3, indicating that VEGFR-3 was also a marker of blood vessels.

### Correlation analysis of podoplanin, LYVE-1, VEGFR-3 and CD31

In 82 paraffin-embedded NSCLC tissues, the mean number of podoplanin^+ ^vessels was 21.5 ± 8.4 (range 7.4–43.6). The mean number of CD31 and VEGFR-3^+ ^vessels was 51.4 ± 11.1 (range 30.0–77.2) and 30.2 ± 16.8 (range 0–46.6), respectively. No substantial association was found between the number of podoplanin^+ ^vessels and CD31^+ ^or VEGFR-3^+ ^vessels (the Spearman rank correlation coefficient *r *= -0.171, *P *= 0.124; *r *= 0.003, *P *= 0.979, respectively). In contrast, high counts of VEGFR-3^+ ^vessels were strongly associated with high CD31^+ ^vessel counts (*r *= 0.331, *P *= 0.002), which showed most VEGFR-3^+ ^vessels were microvalscular vessels not lymphatic vessels.

In addition, in 40 frozen NSCLC tissues, the mean number of LYVE-1^+ ^vessels was 19.9 ± 9.0 (range 5.2–48.0). The mean number of CD31 and podoplanin^+ ^vessels was 52.3 ± 10.9 (range 34.4–71.2) and 22.1 ± 8.1 (range 6.6–44.6), respectively. No substantial association was found between the number of CD31^+ ^vessels and LYVE-1 or podoplanin^+ ^vessels (*r *= 0.009, *P *= 0.957; *r *= 0.059, *P *= 0.717, respectively). In contrast, high counts of LYVE-1^+ ^vessels were strongly associated with high podoplanin^+ ^vessel counts (*r *= 0.525, *P *= 0.001). With the results of morphology above mentioned, LYVE-1+ vessels were most lymphatic vessels, but few of them were micro vessels.

#### VEGF-C expression in NSCLC tissue and its relation to lymph node metastasis

**Figure 3:**
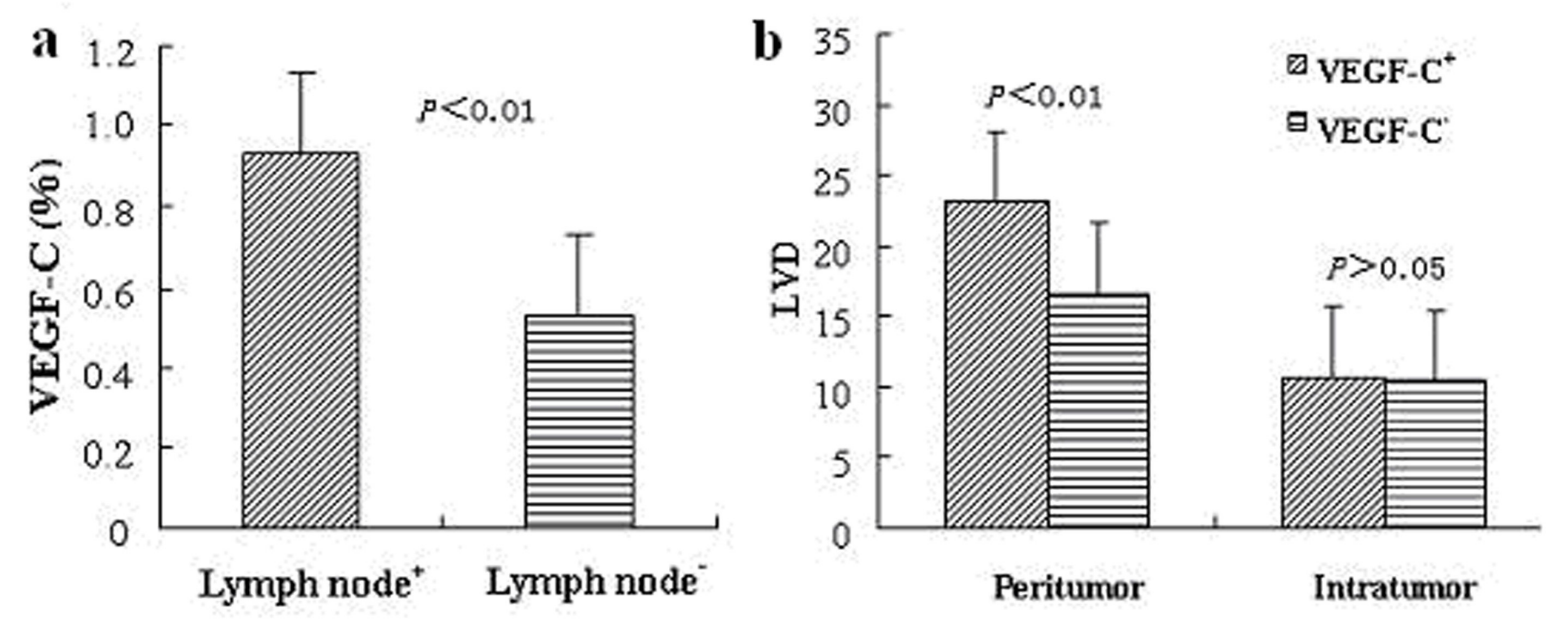
**Prognosis analysis of vegf-c expression**.

Carcinoma VEGF-C expression was classified either as positive *(n *= 61, ≥10% of the carcinoma cells expressed VEGF-C) or negative *(n *= 21, absent expression or expression in < 10% of the carcinoma cells). Among the 82 NSCLC tissues, 61 were VEGF-C positive, 21 were negative, indicating a positive expression rate of 74.4% (61/82). The positive expression rate was significantly higher in the lymph node positive group (93.2%, 41/44) than in the lymph node negative group (52.6%, 20/38) (*P *= 0.000) (Fig. 3a). ptLVD of patients was significantly higher in the VEGF-C positive group than in the VEGF-C negative group (23.1 ± 8.5 vs 15.6 ± 4.2, *P *= 0.000). However, intratumoral lymphatic vessel density (itLVD) values of the two groups showed no significant difference (10.7 ± 5.3 vs 10.4 ± 4.7, *P *= 0.820) (Fig. 3b). According to spearman correlation analysis, VEGF-C expression was positively correlated with ptLVD and lymph node metastasis (*r *= 0.367, *P *= 0.001; *r *= 0.463, *P *= 0.000). Moreover, ptLVD was also positively correlated with lymph node metastasis(*r *= 0.354, *P *= 0.001).

### Associations of Lymphangiogenesis with Clinicopathological Parameters in NSCLC

**Figure 4:**
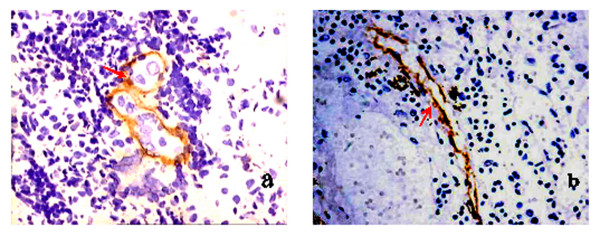
**Double immunostaining with podoplanin and ki-67**.

Double immunostaining with podoplanin and Ki-67 was performed for lymphogenesis analysis (Figure 4). Micro lymphatic vessels were brownish yellow after staining, while the nucleus of the proliferating endothelium cells of the micro lymphatic vessels appeared brownish red (indicated by the red arrow). Cancer embolus was detected in lymphatic vessels(Figure 4a).

The clinic significance was studied by analyzing the peritumoral and intratumoral lymphangiogenesis, various pathological parameters and follow-up data in 82 cases of NSCLC (Table 1). We divided LVD into high LVD Group and low LVD Group according to median. Then the differences was analyzed one by one between ptLVD and itLVD in Age, Gender, Histologic type, Tumor differentiation, Pathologic N stage, Pathologic T stage, Blood vessel invasion (BVI), LVI, Pathologic stage, VEGF-C expression and Ki67%. The mean itLVD was 10.2. No difference was found in any factors between Group high itLVD (n = 46) and Group low itLVD (n = 36) (*P *> 0.05 for all analyses). But ptLVD was different. The median ptLVD was 19.9. No difference was found in LVI, age, gender, the primary tumor size, histologic grade and histologic type between Group high ptLVD (n = 41) and Group low ptLVD (n = 41) (*P *> 0.05). However, high ptLVD Group showed a significant increase than low ptLVD Group in several other clinicopathological parameters, such as lymph node metastasis, LVI, pathologic stage, VEGF-C and Ki67%. Namely, ptLVD was higher in stage III a patients than in stage I and II (*P *< 0.01), in LVI positive than in LVI negative (*P *= 0.004), in lymph node metastasis than in lymph node negative (*P *< 0.01), in VEGF-C positive than in VEGF-C negative (*P *< 0.01) in high Ki67% than in low Ki67%. ptLVD were associated significantly with a higher risk for developing LVI and lymph-node metastasis (*P *< 0.01).

**Table 1:** Association of LVD and LVI with other clinicopathological parameters

**Clinicopathological Parameters**		**Cases**	**ptLVD**	**itLVD**	**LVI** **(-/+)**	**Mean Survival Time ** **(x ± s)**	**median survival time**
Age (y)*	≧55	42	22.1 ± 8.9	10.1 ± 5.1	17/25	1567 ± 138	1658
	<55	40	20.8 ± 7.9	11.2 ± 5.1	21/19	1856 ± 241	1864
Gender	male	63	21.0 ± 7.9	11.2 ± 4.9	27/36	1795 ± 183	1658
	female	19	23.2 ± 9.9	8.7 ± 5.5	11/8	1578 ± 214	1577
Histologic type	Squamous cell	31	19.7 ± 6.4	9.6 ± 4.6	14/17	1664 ± 189	1972
	Adenocarcinoma	41	22.4 ± 9.5	11.0 ± 5.1	20/21	1815 ± 231	1337
	Large cell	10	23.4 ± 9.1	12.4 ± 6.1	4/6	1134 ± 156	1118
Tumor differentiation	Well-moderate	44	21.3 ± 8.6	10.5 ± 5.4	20/24	2085 ± 220	1900
	Poor	38	21.7 ± 8.3	10.8 ± 5.0	18/20	1325 ± 154	1118
Pathologic N stage	N1–2	44	24.2 ± 8.9^#^	10.6 ± 5.4	15/29^#^	943 ± 107^#^	674
	N0	38	18.4 ± 6.7	11.7 ± 4.7	22/16	2725 ± 213	2545
Pathologic T stage	T2–3	51	20.9 ± 8.6	11.4 ± 5.2	21/30	1449 ± 149	1223
	T1	31	22.5 ± 8.0	9.5 ± 4.8	17/14	1875 ± 172	1775
BVI	BVI+	39	23.2 ± 9.8	9.8 ± 4.3	21/18	1321 ± 146	1117
	BVI-	43	19.9 ± 6.6	11.3 ± 5.7	12/21	2083 ± 230	2031
ptLVD*	High(≥19.9)	41	/	12.7 ± 5.6	13/28^#^	1171 ± 153^#^	772
	Low(<19.9)	41	/	12.2 ± 4.9	25/16	2378 ± 224	2057
itLVD*	High(≥10.2)	46	22.9 ± 7.4	/	23/23	1749 ± 229	1577
	Low(<10.2)	36	23.3 ± 6.7	/	15/21	1675 ± 162	1658
LVI	LVI+	46	24.0 ± 9.3^#^	10.9 ± 5.4	/	1212 ± 125^#^	1006
	LVI-	36	18.2 ± 5.8	10.3 ± 4.7	/	2433 ± 245	2123
Pathologic stage	I+II	48	19.4 ± 7.6^#^	10.8 ± 4.9	26/22^#^	2501 ± 202^#^	2115
	III+IV	34	24.5 ± 8.7	10.4 ± 5.4	11/23	800 ± 105	621
VEGF-C	Positive	61	23.1 ± 8.5^#^	10.6 ± 5.0	24/37^#^	1519 ± 173^#^	1117
	Negative	21	16.9 ± 6.0	10.7 ± 5.7	14/7	2232 ± 194	1981
Ki67/%*	High(≥3.56)	50	24.2 ± 9.2^#^	12.9 ± 4.4	21/29^#^	1322 ± 135^#^	1109
	Low(<3.56)	32	17.2 ± 4.8	13.3 ± 5.0	21/11	2431 ± 235	2024

*Cutoff value is median value. ^#^Correlation is statistically significant.

(BVI: Blood vessel invasion, LVI: lymphatic vessel invasion, ptLVD: peritumoral lymphatic vessel density, itLVD: intratumoral lymphatic vessel density, Ki67/%: Ki-67 index of the endothelium cells of the micro lymphatic vessels)

### Associations of LVI with Clinicopathological Parameters

Likewise, the relationship was analyzed between LVI and Age, Gender, Histologic type, Tumor differentiation, Pathologic N stage, Pathologic T stage, Blood vessel invasion, LVI, Pathologic stage, VEGF-C expression and Ki67% (Table 1). Data showed that LVI were significantly associated with lymph-node metastasis, ptLVD, Pathologic stage, VEGF-C expression and Ki67% (*P *< 0.01), but not with itLVD, Pathologic T stage and Blood vessel invasion (BVI).

### Lymphangiogenesis and Prognostic factor in NSCLC

**Figure 5:**
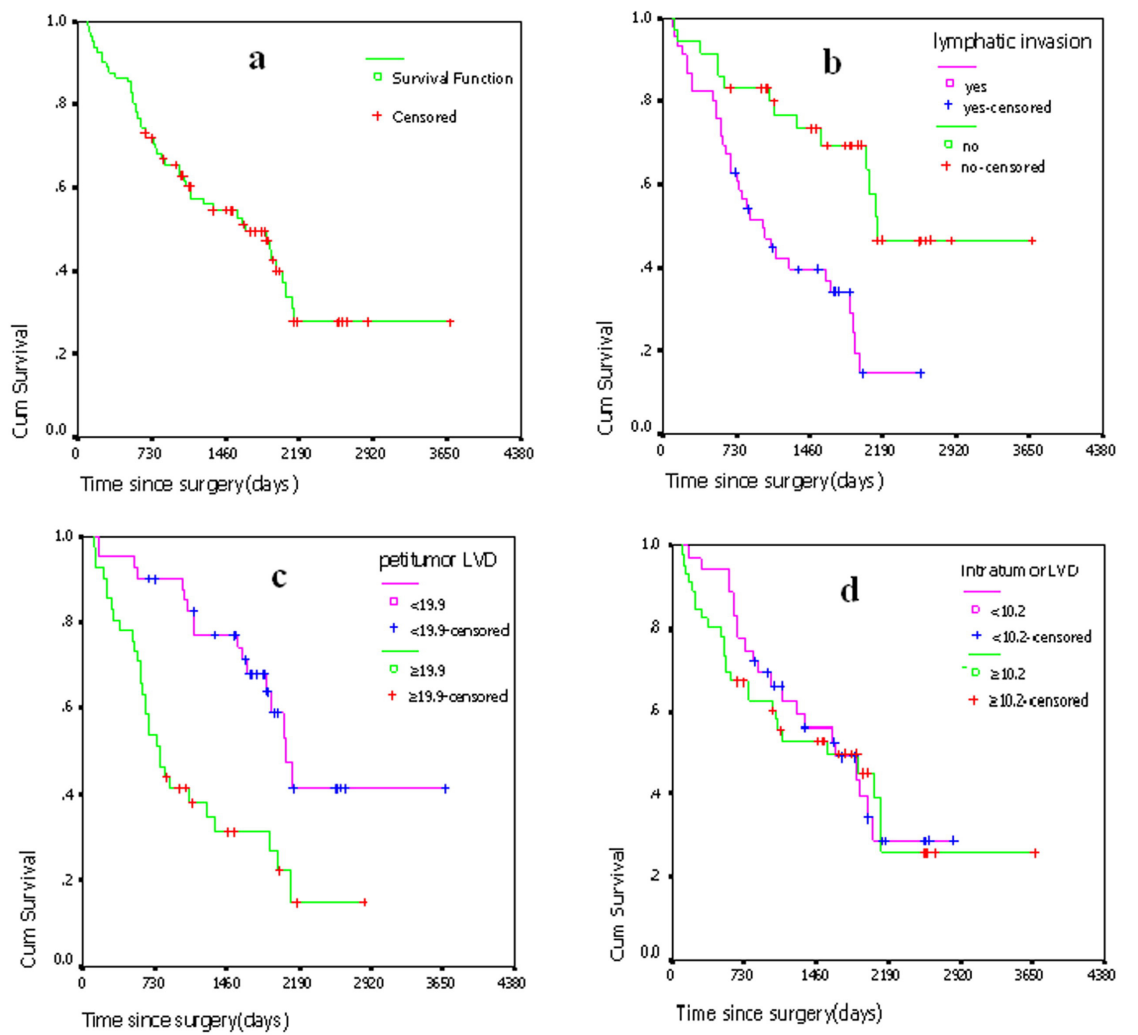
**Survival analysis of clinicopathological parameters**.

The overall survival rate (OS) was 49.3% in 82 NSCLC cases in five years. The median observation time was 1291 days (ranging from 103 to 3680 days). The Kaplan-Meier survival rate curve was showed in Fig 5a. Among it, five year survival rate was 33.5% in LVI^+ ^patients, and 70.0% in LVI^- ^ones. By log-rank test, it was significantly different in survival rate curve in Fig 5b (*P *= 0.0002). Five year survival rate was 31.0% in high ptLVD patients, and 67.6% in low ptLVD ones, showing significant difference in survival rate curve (Fig 5c) (*P *= 0.0001). Five year survival rate was 50.0% in high itLVD patients, and 48.7% in low itLVD ones, showing no significant difference in survival rate curve (Fig 5d) (*P *= 0.7045). In univariate survival analysis, intramural LVD (*P *= 0.719), as well as the patient's age, gender and other clinical and histopathologic parameters had no influence on OS in our collective (*P *> 0.05 for all analyses). A significant difference in OS was found between patients with high and those with low podoplanin^+ ^ptLVD (groups cut off by LVD median) (*P *= 0.0001), with and without LVI (*P *= 0.0002), lymph node status (*P *= 0.0000), pathologic stage (*P *= 0.0000), VEGF-C expression (*P *= 0.0054) and Ki67%(*P *= 0.0001). A multivariate analysis of these individuals was performed using the Cox regression Model. ptLVD, pathologic stage, lymph-node metastasis and Ki67% were independent prognostic parameters for overall survival (*P *= 0.028) (Table 2). Podoplanin positive ptLVD might play important roles in the lymphangiogenesis and progression of NSCLC. Patients with high podoplanin+ ptLVD have a poor prognosis.

**Table 2:** Multivariate analysis of various prognostic factors in patients with NSCLC

	**Univariate**	**Multivariate**		
	
**Prognostic factor**	***P value***	***β***	***P value***	**Relative Risk (95%) CI)**
ptLVD (high/low)	0.0001	0.828	0.003	2.288 (1.182–4.428)
Pathologic stage(I+II/III+IV)	0.0000	1.310	0.003	3.708 (1.581–8.694)
Pathologic N stage (N_0_/N_2–3_)	0.0000	1.218	0.010	3.382 (1.344–8.511)
LVI (-/+)	0.0002	0.714	0.052	2.041 (0.993–4.196)
VEGF-C(-/+)	0.0054	-0.365	0.490	0.694 (0.246–1.958)
Ki67%	0.0012	0.726	0.032	2.067 (1.026–4.161)

(LVI: lymphatic vessel invasion, ptLVD: peritumoral lymphatic vessel density, Ki67/%: Ki-67 index of the endothelium cells of the micro lymphatic vessels)

## Discussion

There are many reports about tumor angiogenesis and poor prognosis in NSCLC. For example, Carcinoembryonic antigen-related cell adhesion molecule 1 (CEACAM-1) has recently been reported to be implicated in cancer development and progression. The elevated CEACAM-1 expression and increased MVD, was an unfavorable prognosis in NSCLC [23]. It has also been reported that high CD34^+ ^MVD and tumour vessel invasion are more closely related to poor survival than the other neoangiogenetic factors in stage IB-IIA NSCLC [24]. In recent years, with the identification of lymphatic endothelial growth factor-C (VEGF-C), VEGF-D and lymphatic endothelial markers including LYVE-1, VEGFR-3 and podoplanin, lymphangiogenesis has become one of the highlights in the field of metastasis in NSCLC. Active lymphangiogenesis is ongoing within sentinel lymph node (SLN) from NSCLC patients, even before metastasis. This lymphangiogenesis may be promoted by upregulation of VEGF121, which may in turn act in part through induction of VEGF-C [25]. Kadota [26] also showed that lymphangiogenesis, specifically Micro-LVD was independently associated with poor prognosis of NSCLC patients. However, these researches can not indicate which LVD status was associated with prognosis of NSCLC patients. What is more, a Meta analysis has been finished [27]. 17 centers provided data for 3200 patients, 2719 of which were included in the analysis. For microvessel density counts obtained by the Chalkley method, the HR for death per extra microvessel was 1.05 (95% CI 1.01–1.09, *P *= 0.03) when analyzed as a continuous variable. For microvessel density counts obtained by the all vessels method, the HR for death per ten extra microvessels was 1.03 (0.97–1.09, *P *= 0.3) when analyzed as a continuous variable. Microvessel density does not seem to be a prognostic factor in patients with non-metastatic surgically treated NSCLC. These conclusions contradict each other. Therefore, the methodology used to assess prognostic factors should be assessed carefully.

Positive correlation was found between the number of podoplanin positive vessels and the number of LYVE-1 positive vessels, while counts of VEGFR-3 positive vessels were correlated with CD31 positive vessel counts. Most of VEGFR-3 vessels, few of LYVE-1 and none of podoplanin positive vessels were blood vessels by observation of light microscope. The results were in accordance with Petri Bono's [28]. In specimens investigated in our study podoplanin expression was restricted to thin-walled lymph vessels with a single endothelial layer. Blood vessels containing red blood cells remained unstained. Podoplanin^+ ^lymph vessels were almost peritumoral, not intratumoral. Lymph vessels could not form in the tumor because of low expression of lymphatic vessel growth factor and high expression of lymph vessel inhibitor factor in the tumor. Furthermore, high interstitial pressure in the tumor was caused with an increase size of lesions [29]. Our research also shows that podoplanin^+ ^ptLVD is associated with lymphatic metastasis, Pathologic stage and Ki67%, and not with histologic type or Tumor differentiation. We presumed that high density of lymph vessels could increase cancer cells to contact with, and invade into lymph vessels, promote lymphatic metastasis and tumor progress. So, podoplanin^+ ^ptLVD is an independent prognostic parameter indeed. Patients with high podoplanin^+ ^ptLVD have a poor prognosis. The result is consistent with the previous research. Saijo [30] showed the recurrence-free survival (RFS) time of patients with high Lymphatic permeation (ly 2) was significantly shorter than that of no Lymphatic permeation (ly 0) patients (*P *< 0.0001), and low Lymphatic permeation(ly 1) patients (*P *= 0.0028). A significant difference in RFS time was also observed between the ly 0 patients and the ly 1 patients (*P *= 0.0025). RFS time of the ly 0 patients was significantly longer than that of the ly 1 plus ly 2 patients (*P *< 0.0001). Saijo only studied Lymphatic permeation (ly) in Lymphangiogenesis and prognosis of patients with NSCLC. Our study further shows that podoplanin^+ ^ptLVD not itLVD is the prognostic parameter. Podoplanin^+ ^ptLVD could also be useful to be a new antitumor target. However, these observations are based only on retrospective analysis of a small case series and further evaluation with a larger number of cases is necessary.

## Conclusion

Podoplanin is the most specific lymphatic endothelial marker. ptLVD and lymph-node metastasis might play important roles in the onset and progression of NSCLC.

## Competing interests

The authors declare that they have no competing interests.

## Authors' contributions

JS conceived of the study, and participated in its design and drafted the manuscript. YW participated in the study design and collected the tissues and carried out the immunoassays. WZ and BZ participated in the immunoassays and performed the statistical analysis. RL helped with the statistical analysis and manuscript drafting. ZC and SZ conceived of the study, and participated in its design and coordination and helped to draft the manuscript.
